# Explaining the heterogeneous scrapie surveillance figures across Europe: a meta-regression approach

**DOI:** 10.1186/1746-6148-3-13

**Published:** 2007-06-28

**Authors:** Victor J Del Rio Vilas, Petter Hopp, Telmo Nunes, Giuseppe Ru, Kumar Sivam, Angel Ortiz-Pelaez

**Affiliations:** 1 Veterinary Laboratories Agency, New Haw, Addlestone, Surrey, UK; 2 Section of Epidemiology, National Veterinary Institute, P.O. Box 8156 Dep., NO-0033 Oslo, Norway; 3 Interdisciplinary Centre of Animal Health Research, Faculty of Veterinary Medicine, Lisbon, Portugal; 4 Reference Centre for Animal Encephalopathies, Institute for Zooprophylaxis, Turin, Italy

## Abstract

**Background:**

Two annual surveys, the abattoir and the fallen stock, monitor the presence of scrapie across Europe. A simple comparison between the prevalence estimates in different countries reveals that, in 2003, the abattoir survey appears to detect more scrapie in some countries. This is contrary to evidence suggesting the greater ability of the fallen stock survey to detect the disease. We applied meta-analysis techniques to study this apparent heterogeneity in the behaviour of the surveys across Europe. Furthermore, we conducted a meta-regression analysis to assess the effect of country-specific characteristics on the variability. We have chosen the odds ratios between the two surveys to inform the underlying relationship between them and to allow comparisons between the countries under the meta-regression framework. Baseline risks, those of the slaughtered populations across Europe, and country-specific covariates, available from the European Commission Report, were inputted in the model to explain the heterogeneity.

**Results:**

Our results show the presence of significant heterogeneity in the odds ratios between countries and no reduction in the variability after adjustment for the different risks in the baseline populations. Three countries contributed the most to the overall heterogeneity: Germany, Ireland and The Netherlands. The inclusion of country-specific covariates did not, in general, reduce the variability except for one variable: the proportion of the total adult sheep population sampled as fallen stock by each country. A large residual heterogeneity remained in the model indicating the presence of substantial effect variability between countries.

**Conclusion:**

The meta-analysis approach was useful to assess the level of heterogeneity in the implementation of the surveys and to explore the reasons for the variation between countries.

## Background

Scrapie is a fatal neurological disease affecting small ruminants. It belongs to the group of diseases known as transmissible spongiform encephalopathies (TSE) that among others include bovine spongiform encephalopathy (BSE) in cattle and Creutzfeldt-Jakob disease (CJD) in humans.

BSE was first detected in 1986 and was shown to spread between cattle by contaminated concentrate [1]. In 1996 it became clear that BSE could be transmitted to humans giving rise to variant CJD [2]. Throughout Europe, scrapie has acquired increased interest because it is considered a potential threat to public health after the successful experimental transmission of BSE to sheep [3] and the likely exposure of sheep to concentrate feed contaminated with the BSE agent [4]. In order to obtain better estimates of the scrapie prevalence throughout the EU, active surveillance for scrapie in small ruminants was introduced in 2002. The surveillance comprised both slaughtered and found-dead animals, namely the abattoir (AS) and fallen stock (FS) surveys respectively [5].

A set of regulations established a regime of procedures that each EU Member State had to follow: i) the sample sizes should be sufficient to detect a prevalence of 0.005% and 0.05% in the populations of slaughtered and found-dead animals, respectively, ii) the target populations were animals older than 18 months of age in both surveys based on dentition checks or any other obvious sign of maturity, and iii) the surveys should be as representative as possible of all breeds, regions and any other characteristic defining some stratification in the standing population. Furthermore, four screening tests for the rapid detection of scrapie in small ruminants were applied consistently throughout the EU during 2003: two Enzyme-Linked ImmunoSorbent Assays (ELISA) tests, a luminescence immunoassay test and a Western-blot based test. The consistent application of these procedures by all Member States should guarantee a certain level of homogeneity in the implementation of the testing across Europe. This would allow fair comparisons between the EU countries with the confidence that the surveys' results do not reflect the effect of artefacts, for example differences in the implementation of the surveys, but true differences in the underlying prevalence of scrapie between countries.

In 2003 the EU Commission Report on the monitoring for the presence of scrapie [6] reported large variation in the frequency estimates of the two surveys between countries. In most of the countries the frequency estimates from the FS were larger than those of the AS. In other countries, however, the FS seemed to detect less scrapie than the AS. This pattern is inconsistent with other works that reported the increased risk of scrapie among the dead on farm animals [7,8] and suggests the occurrence of heterogeneity in the implementation of the surveys between countries; surveys may be reflecting either different situations (e.g. different risks affecting the target individuals by the surveys, tests with different characteristics) or differences in their methodological implementation. There is a need to inform any comparisons between the detected prevalences in the individual surveillance streams.

There have been previous attempts to inform these comparisons. Bird [9] compared the surveillance performance of the two active surveillance sources among EU countries for BSE and scrapie in cattle and sheep respectively. Bird used the tests results from 2001 and 2002, as reported by the EU Commission, to calculate the BSE and TSE rate ratios for each country to describe differences and anomalies in the implementation of the surveys. Bird also produced a EU-pooled measure of the rate ratios between surveillance streams: the median TSE prevalence rate ratio (fallen sheep vs. slaughtered). For the period April to August 2002, Bird reported a rate ratio of 7, which indicated some conformity with the reported 10 times higher prevalence in the fallen stock group for cattle [10]. This increased "efficacy" of the FS is consistent with other works on sheep scrapie [7,8].

Following an approach similar to that of Bird [9], the comparison of some form of frequency ratio between the two surveys throughout the EU, under the standard conditions that apply to the surveys' operations, appears as an adequate methodology to assess the comparability of the scrapie surveillance across Europe. Exploring any differences in the ratios is important because it might help in the understanding of the performance of the surveillance programmes. This might be used to improve the programmes themselves.

The objective of this work is to study the apparent heterogeneity in the behaviour of the surveys across Europe and to investigate the sources of this heterogeneity by taking into account available country-level covariates, potentially explaining methodological differences in the implementation of the surveys, and the effect of the underlying risk in the slaughtered sheep population.

## Results

The test for heterogeneity was significant (Q = 108.6, df = 13, p < 0.0001). Higgins and Thompson [11] statistic suggested that most of the total variation in the estimates of effect was due to the heterogeneity between countries (I^2 ^= 88%, 95% confidence intervals: 82–92).

**Figure 1:**
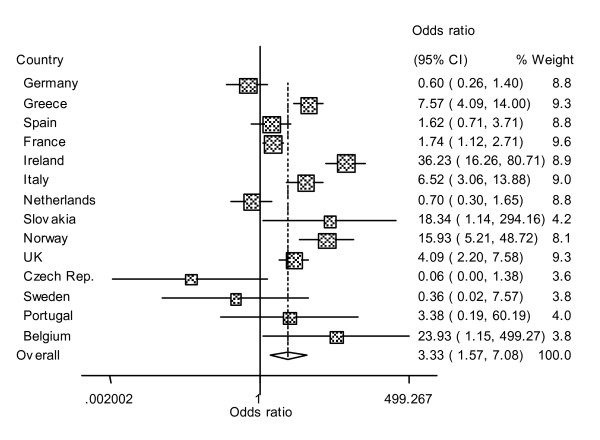
**Forest plot**. Forest plot of the odds ratios (FS/AS) assuming random effects. Only 14 countries after removal of those with zero counts in both surveys. The solid vertical line is showing an odds-ratio of 1 (no effect). The contribution of each country (weights) to the meta-analysis is represented by the area of the box in the plot. The diamond at the bottom shows the overall treatment effect. Weights are derived from the Mantel-Haenszel method.

The forest plot for the random effects meta-analysis of the 14 countries dataset is shown in Figure 1. The pooled effect, the exponentiated logOR, was 3.3 (95% confidence intervals: 1.57–7.08). Germany, The Netherlands, Czech Republic and Sweden showed a better performance of the abattoir survey relative to the fallen stock in detecting cases of scrapie, but the 95% confidence intervals for all included 1. Slovakia, Belgium (although these two with large confidence intervals due to the small numbers), Ireland and Norway showed the best performance of the fallen stock survey in capturing cases of scrapie relative to the abattoir survey.

**Figure 2:**
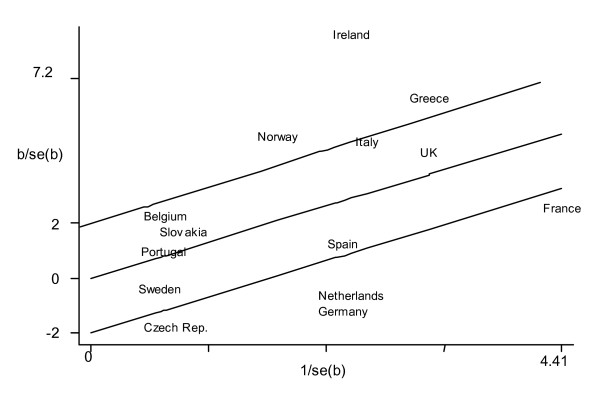
**Galbraith plot**. Galbraith plot. The log-odds ratios (b) divided by their standard errors of the 14 countries (after excluding those countries with zero counts in both surveys) are plotted against the reciprocal of the standard errors (horizontal axis). Solid lines represent the unweighted regression line constrained at 0 with a slope equal to the overall logOR of a fixed effects meta-analysis on our data, and its 95% confidence intervals. The position of the countries in the y-axis indicates their contribution to the Q statistic for heterogeneity. The position of the countries on the x-axis indicates the weight of each country in the meta-analysis.

As illustrated by the Galbraith plot (Figure 2), seven countries (Belgium, Slovakia, Portugal, Sweden, Italy, Spain and the UK) appeared within the 95% confidence interval of the unweighted regression line representing the overall logOR in a fixed effects (assuming no heterogeneity) meta-analysis. Ireland, The Netherlands and Germany were the countries that contributed the most to the between-country heterogeneity given their departure from the 95% confidence interval.

The meta-analysis carried out under the Bayesian framework, on the 18 countries and applying binomial likelihood, allowed the estimation of the uncertainty around the between country heterogeneity (*τ *= 1.73 (95% credible intervals: 0.98, 3.47). The inclusion of the four countries with zero values in both surveys reduced the overall OR (2.61, 95% credible intervals: 0.67, 7.32).

The results of the meta-regression analysis showed that the regression coefficients (*β*) for "test" and "repreAS" included zero in their 95% credible intervals. Their effect on the heterogeneity was not significant.

"repreFS" appeared significantly related to the outcome (*β *= -1.96, 95% credible intervals: -3.82, -0.47). This covariate managed to explain over 18% of the between country heterogeneity (*τ *= 1.42, 95% credible intervals: 0.79, 2.91) when compared to the crude model. Adjusting for the AS-baseline did not appear to have a significant effect in the overall heterogeneity of the model.

## Discussion

We have shown the application of a proven methodology, the meta-analysis framework, to an unusual setting. This has demonstrated the occurrence of great heterogeneity in our measure of choice, the odds ratios between the two surveys, across countries. This can be interpreted as the presence of variability in the way the surveys, across the EU, seem to inform the underlying prevalence of scrapie.

There have been previous attempts to compare the information from the TSE testing results in the EU [9]. This study, however, could not take into account the potential heterogeneity in the surveys' behaviour between countries because of the preliminary nature of the data. Green [12] stated that the detailed description of the design and procedures for case ascertainment should be performed before any comparisons or pooling of estimates was attempted. This is in line with the objective of this paper.

As a by-product, we obtained the overall intervention effect, the pooled OR across Europe. Our results show that scrapie-affected sheep were, on average, 3.3 times more likely to be detected by the fallen stock survey than the abattoir survey throughout Europe. This seems to differ significantly from the expected 10 times higher prevalence from the fallen stock group, by analogy with BSE, as anticipated by the European Scientific Steering Committee (ESSC) [10]. However, the presence of significant heterogeneity prevents one from drawing conclusions on an overall effect. This is true in the case of the meta-analysis and valid too for the meta-regression models where substantial heterogeneity remained.

Kuhnert and Böhning [13] described the occurrence of two types of heterogeneity potentially present in any meta-analysis: the effect heterogeneity and the baseline heterogeneity. The former seems to dominate in our setting after adjusting for the covariates and the baseline variability. Our binomial approach did not return a significant effect of the baseline risks on the model heterogeneity. Binomial approaches are reported to be more appropriate when facing sparse data but they would still require caution in their interpretation [14]. The sensitivity of our results, due to the rarity of our data, to likelihood assumptions was evident when we modelled the data, the 14 countries with the 0.5 continuity correction, under a normal likelihood. In this situation, adjusting for the variability in the baseline risks managed to reduce significantly the model's heterogeneity (*β *= -0.94, 95% credible intervals: -1.88, -0.05).

From the meta-regression approach we can conclude that only the variable "repreFS" appeared to have a significant relationship with the outcome. It also managed to explain some of the between-country heterogeneity in the model. The negative slope, the *β *coefficient for "repreFS" (-1.96), indicates that the greater the proportion of the total adult sheep population sampled by the FS, the lesser the ability of this source in detecting cases of scrapie, relative to that of the abattoir survey. One explanation for this is that some animals that did not fall within the scope of the fallen stock group might have been reported under this surveillance stream introducing an unquantifiable selection bias in the tested population. This would produce a dilution effect, a reduction of the "high risk" nature of this group, reducing the prevalence estimates observed. The fallen stock figures from Germany, for example, might follow this logic. The Food and Veterinary Office (FVO) mission to Germany in November 2002 highlighted the subsidised collection service for the dead on farm animals and the sampling of some fallen stock sheep over 12 months of age [15]. Likewise, the simple observation of Table 1 shows that those countries with smaller samples in the FS seem to detect more scrapie than those with larger samples. We cannot discard the presence of the so called "small-study effects" described in the systematic review literature by which small studies tend to show lower methodological quality and larger effects [16]. Alternatively, the increased risk of scrapie among the slaughtered population, relative to that of the fallen stock group, could also explain the negative logOR of some countries (Table I). If animals from scrapie affected holdings, which had been slaughtered out, were included in the slaughtered population, as culls, this might have increased the risk of scrapie within this group. The 2003 Report [6] stated that, in the context of the eradication measures, a within-holding prevalence of 3% was detected. Similar effects would be observed if the farmers did not submit the found-dead animals, which have shown scrapie-like signs, as fallen stock; in itself another form of selection bias.

**Table 1:** Survey data by country

*Countries*	*r*^*A*^	*n*^*A*^	*r*^*F*^	*n*^*F*^	*LogOR*	*Variance*	*Adult sheep (000)*	*RepreFS*	*RepreAS*	*test*
Belgium	0	2376	2	494	3.18	2.40	146	0.34	1.63	1
Denmark	0	871	0	1320	N/A	N/A	105	1.26	0.83	0.13
Germany	9	20107	13	48616	-0.52	0.19	2637	1.84	0.76	0.91
Greece	49	22564	13	780	2.04	0.10	9042	0.01	0.25	0.43
Spain	19	49921	8	12942	0.48	0.18	23045	0.06	0.22	-
France	46	44641	34	18955	0.55	0.05	8962	0.21	0.50	0.49
Ireland	9	51579	18	2830	3.60	0.17	5907	0.05	0.87	1
Italy	14	35260	13	5011	1.88	0.15	7952	0.06	0.44	0.12
Luxembourg	0	213	0	244	N/A	N/A	7	3.49	3.04	1
Netherlands	45	21095	6	3994	-0.35	0.19	1276	0.31	1.66	0
Austria	0	4225	0	3255	N/A	N/A	304	1.07	1.39	0
Portugal	6	10697	0	243	1.22	2.16	3411	0.01	0.31	1
Finland	0	1990	0	683	N/A	N/A	67	1.02	2.97	0.71
Sweden	2	5175	0	2849	-1.01	2.40	451	0.63	1.15	1
UK	45	72473	13	5113	1.41	0.10	24574	0.02	0.30	0.89
Chzech Rep	1	425	0	2528	-2.88	2.67	103	2.45	0.41	-
Slovakia	1	3923	1	213	2.91	2.00	325	0.07	1.21	-
Norway	5	33519	8	3359	2.77	0.33	928	0.36	3.61	1

*r*^*F *^shows the number of positive samples from the fallen stock, *r*^*A *^shows the number of positive samples from the abattoir survey, *n*^*F *^shows the number of samples tested in the fallen stock and *n*^*A *^the number of samples tested in the abattoir survey. LogOR (log (odds fallen stock/odds abattoir survey)), variance of the logOR (calculated as 1/*r*^*A *^+ 1/*n*^*A *^+ 1/*r*^*F *^+ 1/*n*^*F*^) with 0.5 continuity correction added to all denominators for those countries with one 0 in any of the columns (*r*^*F*^, *r*^*A*^, *n*^*F*^, *n*^*A*^), adult sheep population by country and country-specific covariates inputted into the meta-regression model: "repreFS" (proportion of the adult sheep population sampled by the FS), "repreAS" (proportion of the adult sheep population sampled by the AS) and "test" (proportion of ELISA tests over all samples tested). N/A under the logOR and variance headings show those countries with zero counts in both surveys.

None of the other covariates ("repreAS" and "test") showed a significant effect on the heterogeneity. The data provided by most countries on the use of the rapid tests drove the current parameterisation of this variable as the most comprehensive. Other parameterisations did not produce any different results. Although for nine countries only, "test", as the ratio of ELISA tests used in each survey by each country, did not reduce *τ *either. Our analyses do not capture all the potential variability from the use of the four screening tests across the EU. More data on the use of the screening tests by the countries would be required to improve on the present work.

Other country-specific covariates might inform some of the remaining variability left in the model. However, the amount of information recorded in the European Commission's report [6] was modest. Extra variables to feed into the meta-regression approach, e.g. characteristics related to the operational implementation of the surveys or those that would allow a different parameterisation of the test variability (of special interest would have been those related to the application of the confirmatory tests between the countries), might have explained some further methodological heterogeneity. Genotype information, if available, should always be taken into account in any scrapie-related analyses; such is the importance of this variable in the susceptibility to scrapie. Although genotype data, from an apparent random subset of the AS, was available from [6], only 5 countries genotyped the 500 sheep suggested by the ESSC [10]. We did not believe there was enough data to inform this variable and decided not to use it in our analyses. In the absence of more covariate information, a meta-analysis of the cluster structure of the data, identifying clusters of studies with similar ratios, has been suggested recently to explore the unobserved heterogeneity [13].

The use of a full Bayesian approach is justified by the very same limitations that characterise the traditional weighted regression approach: i) normal likelihood assumption of the measure of effect ii) asymptotic confidence intervals and iii) estimation of *τ *from the data as if it was the true heterogeneity in the population. This was evident after checking the asymmetry of the 95% credible intervals around the posterior estimates for *τ*. This alone would justify the use of a non-asymptotic approach [17].

Other measures of effect could have been chosen: risk ratio or risk difference. However, the mathematical properties of the odds-ratios (with values from 0 to infinity), the lesser heterogeneity displayed by them (when compared to the risk difference) [18] and, for the Bayesian inference, a posterior distribution close to normal even for the sparse data we faced, outweighed any of the other two. Furthermore, the choice of continuity corrections for normal approximations, in our case 0.5, together with the scale of the measure of effect (e.g. a log scale) is likely to have influenced our results [28,31]. For example, the application of the reciprocal of the opposite treatment arm size (FS in our case) as continuity correction [28] returned a pooled OR of 4 (95% confidence intervals: 1.8 – 8.8). The former would also support the use of the binomial likelihood to model our data. This is particularly so when the numbers in the two groups, abattoir and fallen stock, were very imbalanced and the data very sparse [28].

Flat priors were used for all parameters in our Bayesian models. The choice of priors is of great importance, particularly for the variance components [19]. Sensitivity analyses were carried out checking the effect of our choice of priors upon the results. We could not find any significant difference as a result of using different prior distributions.

Caution should also be exercised due to the observational nature of the meta-regression as such. The relationship described by meta-regression does not have the benefit of randomisation to back the interpretation of the results. Any association identified with one country characteristic may in reality reflect a true association with other correlated characteristics, which are likely to be unknown [20].

As a reflection of the increasing number of countries reporting the occurrence of atypical cases of scrapie that produce discordant responses to the established diagnostic tests [21,22], the European Commission's Report [6] clearly stated that all four cases in Sweden and 14 of the 15 from Norway were identified as of the Nor98 type [23]. We did not make any discrimination between the two types of scrapie: the classical and the atypical. Rather, we considered them together for simplicity and to avoid any further layer in our already scarce data. We cannot ignore however the potential contribution of this joint approach to the overall heterogeneity. Multivariate analyses for more than one outcome would be required if the two scrapie types were studied separately [24].

Further studies should look at ways of incorporating more than one year's surveillance data together with the incorporation of more sources of surveillance, namely the statutory reporting and those figures from the eradication measures upon the scrapie affected holdings. This would allow extending this comparison process to the entire surveillance network.

## Conclusion

The meta-analysis of the odds ratios between the two surveys proved a valid and systematic approach to inform the presence of heterogeneity in the operation of the surveys and to identify those countries with the greatest contribution to this variability. The extension of the analysis to a meta-regression allowed the assessment of the effect of the available covariates on the overall heterogeneity. Although substantial heterogeneity remained in the model, unexplained by the scarce data available, there appears to be a need to standardise further the approach to the surveys by the Member States.

## Methods

### Data

Data on the number of sheep tested and confirmed by each surveillance source (AS and FS) were collected from the EU's annual report on the monitoring of transmissible spongiform encephalopathy (TSE) in ruminants in 2003 [6]. Data from 18 European countries were available for the study (Table 1). Data on the screening tests used by each country were not available in the Commission Report nor was possible to obtain them from the Commission due to confidentially issues. Under the collaboration framework of the NeuroPrion Network of Excellence, we requested these data from the National Institutes/Reference Laboratories of all the 18 Member States listed in the report. We did not obtain data from three countries (Czech Republic, Slovakia, and Spain).

### Measures of effect

The number of positive and negative samples for each survey and country can be represented in the form of a 2 × 2 table. Odds ratios (OR) between the fallen stock and the abattoir survey are selected and applied in their logarithmic form: logOR. Table 1 shows the logOR for each country together with their variance.

### Meta-analysis

We used meta-analysis methodologies, and more specifically, meta-regression techniques to explain the variability between countries in this particular context. Meta-analysis is well suited to explore reasons for variation in the observed effect among studies [25]. Other studies have recognised the explanation of the heterogeneity between studies as a logical step and one of increasing importance when conducting meta-analyses [17,26]. If we make the equivalence of countries to studies or centres, the typical study units in traditional meta-analyses, we can run comparisons between countries under the meta-analysis framework.

We first conducted a random effects meta-analysis of the logOR in Stata 8.2 (Stata Corporation, College Station, Texas) to study the presence of heterogeneity. A statistical test of heterogeneity is given in the form of the Q statistic, which has a Chi-square distribution with k-1 (k = number of studies/countries) degrees of freedom [25]. We compared the logORs of the different countries under the assumption that the effects were exchangeable, i.e. the logORs were assumed to be randomly drawn from a population distribution. The random effects model was run using the method of DerSimonian and Laird [27], with the estimate of heterogeneity being taken from the Mantel-Haenszel approach which has been suggested as more adequate for studies with zero events [28]. The traditional random effects model follows

*Y*_*i *_~ *Normal*(*θ*_*i*_, *v*_*i*_) *θ*_*i *_~ *Normal*(*μ*, *τ*^2^)

for *i *= 1,2.... *N *studies, where *Y*_*i *_is the observed effect (the logOR) in the *i*th study/country with variance *v*_*i *_and *θ*_*i *_the study/country specific effects which are sampled from a Normal distribution with mean *μ *and variance *τ*^2^, the between-country heterogeneity.

The quantification of the impact of the between-country variability on the results of the meta-analysis was measured by Higgins and Thompson statistic (I^2^) [11]. Furthermore, forest plots and Galbraith plots [29] (to display graphically the amount of heterogeneity that individual countries contribute) were used to visually help in the interpretation of the data.

To avoid the logOR become undefined for the countries with zero count in either of the surveillance sources [20] we applied a 0.5 continuity correction. Furthermore, to avoid that all the observed effects for a country come from the continuity correction applied, we excluded all those countries with zero counts in both surveys from this part of the analysis. This restricted this analysis to 14 countries after the exclusion of Austria, Denmark, Finland and Luxembourg.

To circumvent the above limitations, the need for continuity corrections and the normality assumption for scarce data, we conducted a second set of analyses following Bayesian methods. We performed the analysis assuming that the observed number of events (samples tested positive) in both groups (abattoir survey (riA
 MathType@MTEF@5@5@+=feaafiart1ev1aaatCvAUfKttLearuWrP9MDH5MBPbIqV92AaeXatLxBI9gBaebbnrfifHhDYfgasaacH8akY=wiFfYdH8Gipec8Eeeu0xXdbba9frFj0=OqFfea0dXdd9vqai=hGuQ8kuc9pgc9s8qqaq=dirpe0xb9q8qiLsFr0=vr0=vr0dc8meaabaqaciaacaGaaeqabaqabeGadaaakeaajugqbiabdkhaYPWaa0baaSqaaiabdMgaPbqaaiabdgeabbaaaaa@3165@) and fallen stock (riF
 MathType@MTEF@5@5@+=feaafiart1ev1aaatCvAUfKttLearuWrP9MDH5MBPbIqV92AaeXatLxBI9gBaebbnrfifHhDYfgasaacH8akY=wiFfYdH8Gipec8Eeeu0xXdbba9frFj0=OqFfea0dXdd9vqai=hGuQ8kuc9pgc9s8qqaq=dirpe0xb9q8qiLsFr0=vr0=vr0dc8meaabaqaciaacaGaaeqabaqabeGadaaakeaajugqbiabdkhaYPWaa0baaSqaaiabdMgaPbqaaiabdAeagbaaaaa@316F@)) followed a binomial distribution [30]

riA~Binomial(piA,niA)i=1,...N countriesriF~Binomial(piF,niF)
 MathType@MTEF@5@5@+=feaafiart1ev1aaatCvAUfKttLearuWrP9MDH5MBPbIqV92AaeXatLxBI9gBaebbnrfifHhDYfgasaacH8akY=wiFfYdH8Gipec8Eeeu0xXdbba9frFj0=OqFfea0dXdd9vqai=hGuQ8kuc9pgc9s8qqaq=dirpe0xb9q8qiLsFr0=vr0=vr0dc8meaabaqaciaacaGaaeqabaqabeGadaaakeaajugqbuaabeqaciaaaeaacqWGYbGCkmaaDaaaleaacqWGPbqAaeaacqWGbbqqaaqcLbuacqGG+bGFcqWGcbGqcqWGPbqAcqWGUbGBcqWGVbWBcqWGTbqBcqWGPbqAcqWGHbqycqWGSbaBcqGGOaakcqWGWbaCkmaaDaaaleaacqWGPbqAaeaacqWGbbqqaaqcLbuacqGGSaalcqWGUbGBkmaaDaaaleaacqWGPbqAaeaacqWGbbqqaaqcLbuacqGGPaqkaeaacqWGPbqAcqGH9aqpcqaIXaqmcqGGSaalcqGGUaGlcqGGUaGlcqGGUaGlcqWGobGtcqqGGaaicqqGJbWycqqGVbWBcqqG1bqDcqqGUbGBcqqG0baDcqqGYbGCcqqGPbqAcqqGLbqzcqqGZbWCaeaacqWGYbGCkmaaDaaaleaacqWGPbqAaeaacqWGgbGraaqcLbuacqGG+bGFcqWGcbGqcqWGPbqAcqWGUbGBcqWGVbWBcqWGTbqBcqWGPbqAcqWGHbqycqWGSbaBcqGGOaakcqWGWbaCkmaaDaaaleaacqWGPbqAaeaacqWGgbGraaqcLbuacqGGSaalcqWGUbGBkmaaDaaaleaacqWGPbqAaeaacqWGgbGraaqcLbuacqGGPaqkaeaaaaaaaa@7C6F@

where, for the *i*^th ^country, niA
 MathType@MTEF@5@5@+=feaafiart1ev1aaatCvAUfKttLearuWrP9MDH5MBPbIqV92AaeXatLxBI9gBaebbnrfifHhDYfgasaacH8akY=wiFfYdH8Gipec8Eeeu0xXdbba9frFj0=OqFfea0dXdd9vqai=hGuQ8kuc9pgc9s8qqaq=dirpe0xb9q8qiLsFr0=vr0=vr0dc8meaabaqaciaacaGaaeqabaqabeGadaaakeaajugqbiabd6gaUPWaa0baaSqaaKqzaeGaemyAaKgaleaacqWGbbqqaaaaaa@31D7@ and niF
 MathType@MTEF@5@5@+=feaafiart1ev1aaatCvAUfKttLearuWrP9MDH5MBPbIqV92AaeXatLxBI9gBaebbnrfifHhDYfgasaacH8akY=wiFfYdH8Gipec8Eeeu0xXdbba9frFj0=OqFfea0dXdd9vqai=hGuQ8kuc9pgc9s8qqaq=dirpe0xb9q8qiLsFr0=vr0=vr0dc8meaabaqaciaacaGaaeqabaqabeGadaaakeaajugqbiabd6gaUPWaa0baaSqaaKqzaeGaemyAaKgaleaacqWGgbGraaaaaa@31E1@ are the number of samples tested in the two surveys and piA
 MathType@MTEF@5@5@+=feaafiart1ev1aaatCvAUfKttLearuWrP9MDH5MBPbIqV92AaeXatLxBI9gBaebbnrfifHhDYfgasaacH8akY=wiFfYdH8Gipec8Eeeu0xXdbba9frFj0=OqFfea0dXdd9vqai=hGuQ8kuc9pgc9s8qqaq=dirpe0xb9q8qiLsFr0=vr0=vr0dc8meaabaqaciaacaGaaeqabaqabeGadaaakeaajugqbiabdchaWPWaa0baaSqaaKqzaeGaemyAaKgaleaacqWGbbqqaaaaaa@31DB@ and piF
 MathType@MTEF@5@5@+=feaafiart1ev1aaatCvAUfeBSjuyZL2yd9gzLbvyNv2CaerbwvMCKfMBHbqedmvETj2BSbqee0evGueE0jxyaibaieIgFLIOYR2NHOxjYhrPYhrPYpI8F4rqqrFfpeea0xe9Lq=Jc9vqaqpepm0xbbG8FasPYRqj0=yi0lXdbba9pGe9qqFf0dXdHuk9fr=xfr=xfrpiWZqaaeaabiGaaiaacaqabeaabeqacmaaaOqaaKqzafGaamiCaOWaa0baaSqaaKqzaeGaamyAaaWcbaGaamOraaaaaaa@398B@ are the underlying probabilities of a positive sample in the two surveys. Furthermore, we express these probabilities, under a logistic regression approach, as

logit(piA)=φilogit(piF)=φi+θiandθi~Normal(μ,τ2)
 MathType@MTEF@5@5@+=feaafiart1ev1aaatCvAUfKttLearuWrP9MDH5MBPbIqV92AaeXatLxBI9gBaebbnrfifHhDYfgasaacH8akY=wiFfYdH8Gipec8Eeeu0xXdbba9frFj0=OqFfea0dXdd9vqai=hGuQ8kuc9pgc9s8qqaq=dirpe0xb9q8qiLsFr0=vr0=vr0dc8meaabaqaciaacaGaaeqabaqabeGadaaakeaajugqbuaabaqacmaaaeaacqGGSbaBcqGGVbWBcqGGNbWzcqGGPbqAcqGG0baDcqGGOaakcqWGWbaCkmaaDaaaleaacqWGPbqAaeaacqWGbbqqaaqcLbuacqGGPaqkcqGH9aqpiiGacqWFgpGzkmaaBaaaleaacqWGPbqAaeqaaaqcLbuabaaabaaabaGaeiiBaWMaei4Ba8Maei4zaCMaeiyAaKMaeiiDaqNaeiikaGIaemiCaaNcdaqhaaWcbaGaemyAaKgabaGaemOrayeaaKqzafGaeiykaKIaeyypa0Jae8NXdyMcdaWgaaWcbaGaemyAaKgabeaajugqbiabgUcaRiab=H7aXPWaaSbaaSqaaGqaciab+LgaPbqabaaajugqbeaacqqGHbqycqqGUbGBcqqGKbazaeaacqWF4oqCkmaaBaaaleaacqWGPbqAaeqaaKqzafGaeiOFa4NaemOta4Kaem4Ba8MaemOCaiNaemyBa0MaemyyaeMaemiBaWMaeiikaGIae8hVd0MaeiilaWIae8hXdqNcdaahaaWcbeqaaiabikdaYaaajugqbiabcMcaPaaaaaa@703D@

where, for the *i*^th ^country, *ϕ*_*i *_is the estimated log-odds of a positive sample in the abattoir survey (called the "baseline risk"), *θ*_*i *_is the logOR between the two surveys (also called the "treatment effect") and *μ *and *τ*_2 _have the same interpretation as in equation (1). A normally distributed, non-informative prior is regularly assigned to *μ *(Normal (0, 10000)) and a Uniform prior, non-informative, to *τ*^2 ^(Uniform(0,10)). Sensitivity analysis, to the choice of priors, is regularly conducted by changing the prior distributions for *τ*^2^, for example to a Normal(0, 10000) zero truncated. We assumed independent baselines between countries dismissing thus any relationship or overall distribution across the underlying risks. We allocated a non-informative prior to the underlying baseline risks *ϕ*_*i*_: Normal(0, 100). An important advantage of the binomial approach is that we can use all data points, the 18 countries, regardless of the presence of zero counts in the two surveillance sources. Another important benefit from the use of a Bayesian approach is the estimation of the credible intervals around the point estimate of *τ*^2 ^as this is now treated as a random variable with uncertainty in its estimation [31].

### Meta-regression

In the presence of heterogeneity, pooled/overall estimates of effect should not be reported. The aim should shift to explain the causes of variability between studies. Meta regression aims to explain, by the inclusion of country-specific covariates, the heterogeneity of the effects between countries. Some of this variability may have come from the different screening tests used by the countries. A homogeneous situation would be that where all countries use the same test in both surveys. Heterogeneity would appear as soon as, at least, one country uses a different test, with different characteristics, in, at least, one of the surveys. In our setting, we did not have complete data on both surveys from all countries. Furthermore, some countries used more than one test in either survey making difficult to inform this parameter in the model in such a way that, for the majority of countries, the variable captured meaningful variability. We have modelled the test variability between countries ("test") as the proportion of ELISA tests over all samples tested, used by each country. We chose this parameterisation because it was the one for which more data were available to inform both surveys for the largest number of countries. Furthermore, we modelled the proportion of the country's adult sheep population sampled by the fallen stock ("repreFS") and the proportion of the adult sheep population sampled by the abattoir survey ("repreAS") as measures of representativeness.

The heterogeneity between studies can also be due to the baseline risk, in our case the event rate in the abattoir survey. Differences in the genotype distribution and/or the survivorship of the slaughtered population between countries might result in different risks in the abattoir survey. Thompson et al. [17] and Arends et al. [32] emphasized the influence of the underlying risk of patients in the different trials/studies upon the treatment benefit we observe. Warn et al. [31] defined the underlying risk as a trial-level summary of individual-level characteristics that would represent the health status of the population sampled by the study. We have assessed the relationship of the logOR (FS/AS) with the baseline risk (AS) to account for the potential variability in the risks of the slaughtered populations between countries on the overall heterogeneity.

The combination of a structural dependence between the outcome and our baseline and the fact that both are estimated from a finite sample (they are measured with error) present problems associated with regression to the mean, also known as regression dilution bias. Any of these events could lead to a biased slope in an ordinary weighed least squares regression line [32]. The application of the Bayesian approach facilitates the investigation of the relationship between the true baseline risk and the true measure of effect as it models the relationship between the true parameters rather than observed values.

We extended our previous equations to allow for the covariates and the baseline, following Sharp and Thompson [26], into the model

θi=θi∗+βxi+γ(φi−φ¯)i=1,...N countriesθi∗~N(μ,τ2)
 MathType@MTEF@5@5@+=feaafiart1ev1aaatCvAUfKttLearuWrP9MDH5MBPbIqV92AaeXatLxBI9gBaebbnrfifHhDYfgasaacH8akY=wiFfYdH8Gipec8Eeeu0xXdbba9frFj0=OqFfea0dXdd9vqai=hGuQ8kuc9pgc9s8qqaq=dirpe0xb9q8qiLsFr0=vr0=vr0dc8meaabaqaciaacaGaaeqabaqabeGadaaakeaajugqbuaabaqaciaaaeaaiiGacqWF4oqCkmaaBaaaleaacqWGPbqAaeqaaOGaeyypa0Jae8hUde3aa0baaSqaaiabdMgaPbqaaiabgEHiQaaakiabgUcaRiab=j7aIjabdIha4naaBaaaleaacqWGPbqAaeqaaOGaey4kaSIae83SdCMaeiikaGIae8NXdy2aaSbaaSqaaiabdMgaPbqabaGccqGHsislcuWFgpGzgaqeaiabcMcaPaqcLbuabaGaemyAaKMaeyypa0JaeGymaeJaeiilaWIaeiOla4IaeiOla4IaeiOla4IaemOta4KaeeiiaaIaee4yamMaee4Ba8MaeeyDauNaeeOBa4MaeeiDaqNaeeOCaiNaeeyAaKMaeeyzauMaee4CamhabaGae8hUdeNcdaqhaaWcbaGaemyAaKgabaGaey4fIOcaaKqzafGaeiOFa4NaemOta4KaeiikaGIae8hVd0MaeiilaWIae8hXdqNcdaahaaWcbeqaaiabikdaYaaajugqbiabcMcaPaqaaaaaaaa@6B0A@

where, for the i^th ^study, θ * is the effect adjusted for the underlying risk and *x *the value of the covariate, *β *and *γ *the unconstrained regression coefficients and φ¯
 MathType@MTEF@5@5@+=feaafiart1ev1aaatCvAUfKttLearuWrP9MDH5MBPbIqV92AaeXatLxBI9gBaebbnrfifHhDYfgasaacH8akY=wiFfYdH8Gipec8Eeeu0xXdbba9frFj0=OqFfea0dXdd9vqai=hGuQ8kuc9pgc9s8qqaq=dirpe0xb9q8qiLsFr0=vr0=vr0dc8meaabaqaciaacaGaaeqabaqabeGadaaakeaadaqdaaqcLbuabaacciGae8NXdygaaaaa@2F2C@ the mean underlying risk across countries. A non-informative prior distribution, a normal with large variance, must be given to *β *and *γ*. These two parameters, *β *and *γ*, are the ones, together with the between-country standard deviation (*τ*), of principal interest.

We incorporated each covariate, and the baseline, into the model in a univariate fashion and checked the significance of their regression coefficients, whether their 95% credible intervals included the value zero, and their impact on the value of *τ*.

We ran the binomial model in WinBUGS 1.4.1 [33] to derive, from the marginal posterior distributions of our parameters of interest, medians, standard deviations and 95% credible intervals. We checked the Gelman-Rubin plots for convergence.

## Authors' contributions

VJDRV conceived the study, carry out the statistical analyses and drafted the manuscript. PH conceived the study and critically revised the manuscript. TN, GR, SKS and AOP critically revised the manuscript. All authors read and approved the final manuscript.
